# Binding in working memory and frontal lobe in normal aging: is there any similarity with autism?

**DOI:** 10.3389/fnhum.2015.00090

**Published:** 2015-03-05

**Authors:** Grégory Lecouvey, Peggy Quinette, Grégoria Kalpouzos, Bérengère Guillery-Girard, Alexandre Bejanin, Julie Gonneaud, Ahmed Abbas, Fausto Viader, Francis Eustache, Béatrice Desgranges

**Affiliations:** ^1^INSERM, U1077Caen, France; ^2^UMR-S1077, Université de Caen/Basse-NormandieCaen, France; ^3^UMR-S1077, Ecole Pratique des Hautes EtudesCaen, France; ^4^UMR-S1077, Caen University HospitalCaen, France; ^5^Aging Research Center, Karolinska Institute and Stockholm UniversityStockholm, Sweden; ^6^Department of Neurology, Caen University HospitalCaen, France

**Keywords:** aging, binding, executive functions, processing speed, brain metabolism, frontal lobes

## Abstract

Some studies highlight similarities between Autism Spectrum Disorder (ASD) and healthy aging. Indeed, the decline in older individuals’ ability to create a unified representation of the individual features of an event is thought to arise from a disruption of binding within the episodic buffer of working memory (WM) as the same way as observed in ASD. In both cases, this deficit may result from an abnormal engagement of a frontohippocampal network. The objective of the present study is to identify both cognitive processes and neural substrates associated with the deficit of binding in WM in healthy aging. We studied the capacity of binding and the cognitive processes that might subtend its decline in 72 healthy participants aged 18–84 years. We examined the behavioral data in relation to the changes in brain metabolism associated with the age-related decline in a subgroup of 34 healthy participants aged 20–77 years using the resting-state [^18^F] fluorodeoxyglucose positron emission tomography (^18^F-FDG PET). Forward stepwise regression analyses showed that the age-related decline in binding was partially explained by a decline in inhibition and processing speed. PET correlation analyses indicated that metabolism of the frontal regions, anterior and middle cingulate cortices is implicated in this phenomenon. These data suggest that executive functions and processing speed may play a crucial role in the capacity to integrate unified representations in memory in aging. Possible implications are discussed in ASD.

## Introduction

Episodic memory refers to a memory system that stores memories of personal events located in space and time, associated to self-referential and contextual environment. Encoding and recollecting episodic memories imply to create multimodal traces that will be achieved by binding mechanisms. This very complex memory system is impaired in Autism. There is growing evidence that individuals with high-functioning Autism Spectrum Disorder (ASD) show impaired contextual memory (Bowler et al., 2008; Gaigg et al., 2008; Souchay et al., 2013b) and relational memory (Southwick et al., 2011; Maister et al., 2013). In addition, other studies reported difficulties in the capacity to mentally re-experience or recollect the personal event with all associated phenomenological details (Bowler et al., 2000; Souchay et al., 2013a). Overall, these results lead some authors to compare these memory difficulties in ASD with the memory decline observed in healthy aging (Bowler et al., 2004). This “aging analogy” refers to difficulties in processing complex stimuli and binding together the different features that define an episodic memory trace in Working Memory (WM; Baddeley, 2000). WM is a memory system comprising two slave systems (i.e., the phonological loop and the visuospatial sketchpad) responsible for the processing and temporary storage of information useful for understanding, learning and reasoning (Eustache and Desgranges, 2008). This suggests a dysfunction of the episodic buffer that refers to a working space where information from different sources is bound into a unitary representation and stored as a multimodal one for several seconds in WM. It is conceptualized as an interface between the central executive which supervises and coordinates the information supplied by the slave systems and long-term memory (Baddeley et al., 2011). The *episodic* label refers to the hypothetical role it plays during encoding and conscious recollection of events in episodic memory, whereby it integrates information that becomes accessible to conscious awareness (Eustache and Desgranges, 2008).

Indeed, it has been suggested that an associative deficit disturbing the creation of associations (and equivalent to a binding deficit) is one of factors contributing to diminished episodic memory both in older adults (Naveh-Benjamin, 2000; Naveh-Benjamin et al., 2003) and in ASD (Bowler et al., 2010; Maister et al., 2013). Using a task where individuals were shown 30 colored objects within a 7 × 7 array for 90 s and had to immediately recognize these objects and their location, Chalfonte and Johnson (1996) were the first to show that while memory for individual features (objects, colors and locations) is preserved in aging, memory for bound features (e.g., object + color and object + location) suffers from an age-related decline. These results in support of a general age-related decline in binding have since been replicated (Mitchell et al., 2000a,b; Plancher et al., 2010). Interestingly, the same task was recently used by Bowler et al. (2014) in a group of adults with ASD and the same pattern of performances was found: relatively undiminished performances for individual features contrasting with significant difficulties in combination conditions. These results suggest that processing steps from perceptual analysis to item-based memory are broadly spared in contrast with an impairment of the binding process *per se*.

The cognitive substrates of binding have been studied in various populations. Baddeley and Wilson (2002) found positive correlations between immediate prose recall, a task that involves the binding of verbal information into meaningful units, and both executive (shifting and inhibition processes) and intellectual (Wechsler Adult Intelligence Scale; Wechsler, 1987) performances, in densely amnesic patients. In the context of ASD and aging, binding difficulties are thought to result from difficulties in the executive processes (Maister et al., 2013). For instance, Mitchell et al. (2000b) hypothesized that the age-related binding decline stems from an age-related decline in the executive processes involved in WM (e.g., shifting of attention from one stimulus to another, implementation and maintenance of a cumulative rehearsal strategy). To test their hypothesis, they asked young and older individuals to recognize objects, locations or object-location combinations after an 8-s unfilled interval. While young and older adults performed at the same level when it came to recognizing individual features or targets in the combination condition, the older adults produced more false alarms in the latter condition. They were also slower than the young adults at rejecting lures in the combination condition, either in an attempt to improve their accuracy, or because of an age-related processing speed decrement which has been suggested to account for some of the age-related differences reported in measures of cognition (Salthouse, 1996). Mitchell et al. concluded that the older individuals may have experienced difficulties using an efficient rehearsal strategy, possibly owing to a decline in reflective and executive processes. Following up on this study, Naveh-Benjamin found that older individuals performed more poorly during the incidental encoding of associative information (i.e., learning a list of word-nonword pairs and recognizing them after a 90-s interval) and exhibited an even greater deficit in the use of strategic behavior when they directed their attention to the relevant associative information (Naveh-Benjamin, 2000). In other words, older adults exhibit a greater deficit when strategic processing involving executive functions (inhibition of irrelevant information, shifting between strategies) is required.

Neuroimaging studies in young adults have revealed the involvement of a frontohippocampal network during feature binding (Mitchell et al., 2000a), and several hypotheses have been put forward as to the respective roles of these two structures. Damage to the hippocampus is typically associated with massive impairment of long-term episodic memory, possibly owing to a disruption of binding (Cohen and O’Reilly, 1996; Reinitz et al., 1996; Quinette et al., 2006; Braun et al., 2008; Finke et al., 2013). Although the hippocampus is traditionally associated with episodic memory, recent studies have shown that it is also engaged over short time spans (Ranganath et al., 2005; Piekema et al., 2006; Olsen et al., 2012). More precisely, the hippocampus may receive information from multiple cortical regions and automatically bind it together (Vargha-Khadem et al., 1997; Eichenbaum, 2000; Baddeley et al., 2010; Opitz, 2010; Olsen et al., 2012). Some authors have suggested that the hippocampus is involved in actively maintaining associations with spatial information, but not with other types of information, although this issue has yet to be explored (Mitchell et al., 2000a; Piekema et al., 2006). Finally, others have conjectured that the hippocampus actively forms and consolidates long-term memory traces of associations immediately after their creation and plays a role in the transition from WM to long-term memory rather than in WM *per se* (Piekema et al., 2006; Quinette et al., 2006; Allen et al., 2014).

Concerning frontal involvement, two studies have provided evidence that the medial prefrontal cortex is more active when young adults have to remember a combination of features, rather than individual ones (Mitchell et al., 2000b; Prabhakaran et al., 2000). These frontal areas may underlie the reflective processes that allow for the coordination of strategies to control the coactivation of features and the length of time they remain coactive, in order to permit the short-term maintenance and manipulation of the integrated features or to permit the integration itself (Mitchell et al., 2000a; Prabhakaran et al., 2000; Koechlin and Summerfield, 2007).

Mitchell et al. (2000a) have examined the brain substrates of the age-related decline in effortful binding. They used functional MRI to explore differences in activation between younger and older adults during the recognition of object-location associations. Their findings suggested that an age-related dysfunction of the left anterior hippocampus accounts for the difficulty that older people have with feature binding in WM. The authors also found that while the prefrontal cortex subtended the retrieval of bound features in young adults, this was not the case for older adults, who performed more poorly (Mitchell et al., 2000a). This result is in line with reports of frontal lobe disruption with advancing age in neuroimaging studies (for reviews, see Raz and Rodrigue, 2006; Kalpouzos et al., 2009a) even if other studies suggest that increase in frontal lobes activation during memory tasks may reflect the use of compensatory strategies in response to a structural decline (Persson et al., 2006; Miller et al., 2008). In summary, these results suggest that the integrity of the frontohippocampal network is critical for feature binding.

The present study aimed at exploring the capacity of binding in healthy aging by combining neuropsychological and neuroimaging approaches. We had three main objectives. First, previous studies had suggested an age-related decline of binding on tasks featuring intervals that were too long to allow for the formation and storage of associations to be dissociated. Our study was therefore designed to explore age-related effects in a feature-binding task with a very short 1 s interval (Quinette et al., 2006), in order to focus on the formation stage. Second, although the disruption of effortful binding is thought to stem from a reduction in processing speed and a decline in executive processes (shifting, inhibition, updating), the respective contributions of these cognitive functions has never been fully assessed. In order to gain a clearer understanding of their contribution to the age-related binding decline, we therefore conducted forward stepwise regression analyses. Finally, neuroimaging findings have suggested that the disruption of a frontohippocampal network could be responsible for the age-related decline in binding (Mitchell et al., 2000a). In our study, where we focused on effortful binding which is more conducive to frontal lobe involvement (since participants had to mentally combine verbal and spatial features), we therefore expected an age-related reduction in frontal lobe metabolism to mainly account for the age-related decline in strategic binding.

## Material and methods

### Participants

This protocol was approved by the regional ethics committee (CPP Nord-Ouest III). The participants all gave their written informed consent.

#### Cognitive data sample

A total of 72 healthy individuals aged 18–84 years (mean age = 45.75 ± 18.83 years) underwent the cognitive assessment. As the participants were homogeneously distributed across the age groups, age was treated as a continuous variable. Descriptive data are reported in Table 1. All participants stated that they were in good health in a health questionnaire. They did not report any history of neurological or psychiatric conditions, head trauma, or alcohol and drug abuse. Individuals aged 50 years or above were screened for general cognitive impairment on the Dementia Rating Scale (Mattis, 1976). All had normal performances for their age and education level. Each participant had received at least eight full years of education. Because of changes in the French education system over the years, we assessed education level using a composite index (see Gonneaud et al., 2011) that took into account both the participants’ years of schooling and their vocabulary level (assessed with Part B of the Mill Hill test; Deltour, 1993). There was no significant correlation between age and this composite index (*r* = 0.07, *p* = 0.55).

**Table 1:** **Characteristics of the two samples**.

	Whole sample	Imaging subsample
Number	72	34
Women/Men	40/32	19/15
Age (years): mean ± SD	45.75 ± 18.83	46.79 ± 18.82
Age range	18–84	20–77
Mean years of education ± SD	12.47 ± 2.91	12.82 ± 2.73
Mean Mill Hill vocabulary score /44 ± SD	33.97 ± 4.98	35.09 ± 4.36

#### Imaging subsample

Of the 72 participants included in the cognitive sample, 34 individuals aged 20–77 years, all right handed except for three who were ambidextrous, underwent structural T1-weighted MRI and [^18^F]fluorodeoxyglucose positron emission tomography (^18^F-FDG PET) scans. Descriptive data are reported in Table 1. The subsample did not differ from the original sample in terms of age, sex ratio or education level. The T1-, T2- and/or FLAIR-weighted MRI scans were normal for all participants, with no significant white-matter hyperintensities on the T2/FLAIR-weighted images (see Kalpouzos et al., 2009a, for further details). As with the whole group, there was no significant correlation between age and the composite index of education level (*r* = −0.11, *p* = 0.53).

### Procedure

The protocol comprised an assessment of binding in WM, executive functions (shifting, inhibition and updating), manipulation of information in WM and processing speed, divided in two sessions separated by a one-week interval. Binding was assessed during the first session.

### Materials

#### Binding

**Figure 1:**
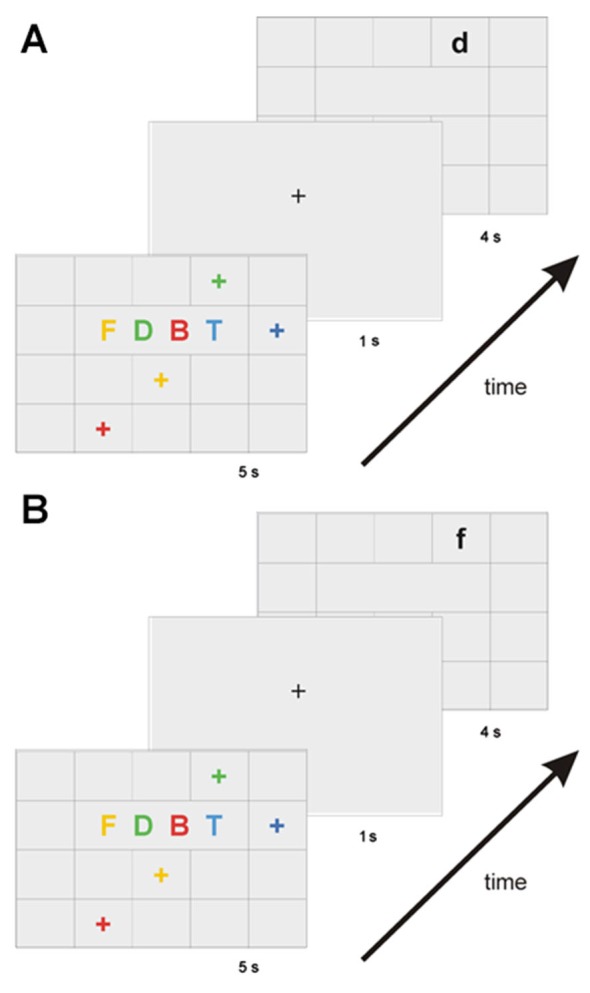
**Diagram of the working memory binding task**. After the binding processing (matching letter with location according to color), participants had to memorize the integrated information for 1 s. Two types of stimuli were presented: the target type **(A)**, when the letter was in the right location, or the lure type **(B)**, when the letter was in the wrong place.

We assessed the association of multimodal information by means of a WM binding task that had already been used in several studies in our laboratory, and which measures the ability to associate verbal and spatial features (Mitchell et al., 2000a,b; Prabhakaran et al., 2000; Quinette et al., 2006, 2013; Hainselin et al., 2011; see Figure 1). The task was presented on a computer using SuperLab Pro software, which also recorded responses. In the learning phase, stimuli consisted of four colored uppercase consonants, displayed for 5 s in the center of a 5 × 4 grid, and four colored crosses placed randomly in the remaining 16 squares. Participants were asked to mentally match each consonant with the location represented by the cross of the same color. After a 1-s retention interval, a black letter was displayed in a specific square in the grid, and participants had 4 s to determine whether the letter was in the same place as it had been matched in the learning phase. The “L” and “D” keys on the keyboard randomly corresponded across participants to “yes” and “no”. Individuals were asked to answer as quickly as possible, though giving priority to accuracy. For half the stimuli, the letter was in the right location (target type), and for the other half, the letter was in the wrong place (i.e., a place that was initially matched with another letter, lure type). The task was divided into two sessions of 10 trials, and the score was the number of correct answers (accepted targets and rejected lures) out of 20 trials.

#### Other cognitive function assessments

In order to investigate whether the effect of age on binding was mediated by an effect of age on other cognitive functions, the neuropsychological assessment included tests of cognitive functions that are thought to play a role in binding. The neuropsychological battery comprised assessments of executive functions (shifting, inhibition, updating), the central executive of WM, and processing speed.

##### Executive functions

We administered a set-shifting test (Mayr and Kliegl, 2000) in which participants alternated between two different tasks every four trials. They were given 16 words accompanied by a sign that changed every four trials. When this sign was a heart, they were asked to make a living/nonliving judgment for each word. When the sign was a cross, they were asked to decide whether the words represented something bigger or smaller than a soccer ball. The measurement was the switch cost, that is, the difference between the time it took for participants to answer in situations where there was a shift from a task to the other one and in situations where there was none.

Inhibition process was assessed using the Stroop Color and Word Test (Stroop, 1935). Participants had to name colored dots (e.g., blue, red, green) in the first condition; to read out the name of common colors printed in black in the second condition; and to name the color in which the names of colors were printed as quickly as possible (e.g., “red” printed in blue, “green” printed in red, etc.) in the last condition. The inhibition score was the time it took to participants to complete the third condition (i.e., interference score).

The running span task (Quinette et al., 2003; adapted from Morris and Jones, 1990) assessed updating. Participants were orally provided with 16 consonant strings of variable length (4, 6, 8 or 10 letters) without any prior information about their length, and were required to recall the last four items in each string, in the same order as they had been provided. The updating score was the total number of successfully recalled strings.

##### Central executive of working memory

The central executive of WM was assessed using the backward digit span (Wechsler Adult Intelligence Scale; Wechsler, 1987). The score was the length of the correct sequence containing the greatest number of items.

##### Processing speed

The BAMS-T (Lahy, 1978) was administered to measure processing speed. Participants were provided with a sheet of paper filled with rows of eight different symbols and asked to cross out every instance of one of the three target symbols printed at the top of the sheet. The processing speed score was the total number of crossings-out per second.

### Imaging acquisition

T1-weighted MRI images, used to coregister the PET images, were acquired on a General Electric 1.5-tesla Sigma Advantage echoplanar imaging device. There were 128 adjacent axial slices parallel to the anteroposterior commissure (AC-PC line), with a slice thickness of 1.5 mm and in-plane resolution of 0.94 × 0.94 mm. A spoiled gradient-echo sequence was used, with a repetition time (TR) of 10.3 ms, an echo time (TE) of 2.1 ms, a field of view (FOV) of 24 × 18 cm, and a matrix of 256 × 192. The standard correction for field inhomogeneities was applied.

The regional distribution of radioactivity was followed using an ECAT Exact HR+ PET scanner with a resolution of 4.6 × 4.2 × 4.2 mm and an axial FOV of 158 mm. Participants were scanned with their eyes closed, in a dark and quiet room (resting state). Their head was immobilized in a headrest following the orbitomeatal line. The radiotracer was injected via a catheter inserted into a vein in the arm. Transmission scans were obtained with a 68 Ge source. At Time 0, 3–5 mCi (111–185 Mbq) of ^18^F-FDG were injected as a bolus, and a 10-min data acquisition session began 50 min post-injection. Full 3D volume acquisition allowed for the reconstruction of 63 planes, with a voxel size of 2.2 × 2.2 × 2.43 mm.

### Behavioral data analysis

We conducted Pearson’s correlations between (i) binding performance and age, to assess the effect of age on binding; (ii) other cognitive scores and age, to determine whether other cognitive functions were sensitive to age; and (iii) binding scores and other cognitive scores, to determine whether binding performance was linked to other cognitive abilities. We then conducted forward stepwise regression analyses on the binding scores, including all the cognitive scores that correlated with binding and with age, in order to identify the variables that best explained the interindividual variability in binding performance. This model also allowed us to select variables for the subsequent imaging analyses.

### Neuroimaging data analyses

#### Preprocessing of the images

All imaging data were preprocessed and analyzed using statistical parametric mapping software (SPM5; Wellcome Department of Cognitive Neurology)^1^ implemented in Matlab (MathWorks, Sherborn, MA, USA). The T1-weighted MRI images were preprocessed using the voxel-based morphometric procedure (VBM), with the VBM5.1 toolbox, which corresponds to a unified segmentation approach (Ashburner and Friston, 2005). PET images were first corrected for partial volume effects (PVEs) using PMOD software (Quarantelli et al., 2004), for an optimized voxel-based method. This software allowed us to correct for gray-matter (GM) signal loss owing to spill-out onto non-GM tissues, and for GM signal increase caused by spill-in from adjacent white matter. Every reconstructed PET image was coregistered to the corresponding MRI image and spatially normalized to the MNI template by adopting the normalization parameters obtained from the unified segmentation procedure applied to the MRI images. These PET images were resampled to a voxel size of 1 × 1 × 1 mm. The resulting PET images were then divided by their individual vermis ^18^F-FDG uptake values to control for individual variations in global PET measures, following a procedure already used in our laboratory (for details, see Mevel et al., 2007). A 14-mm isotropic Gaussian filter was applied to the images to smooth them in order to compensate for interindividual differences and maximize the signal-to-noise ratio. Smoothed and scaled PET data were masked in order to keep only GM voxels of interest for further analyses. A statistical threshold of *p(uncorrected)* < 0.001 and cluster extent *k* > 150 voxels was used to achieve a corrected statistical significance of *p* < 0.05 determined by Monte-Carlo simulation (see program AlphaSim by D.Ward).

#### Negative correlations between ^18^F-FDG uptake and age (mask)

Negative correlations were conducted across the 34 participants by performing a multiple regression in SPM5 between preprocessed PET images and age. The result of this analysis was used as a mask for the forthcoming correlations analyses between ^18^F-FDG uptake and binding in order to constrain results within clusters for which age-related decrease in metabolism was observed.

#### Positive correlations and partial correlations (with cognitive functions partialed out) between ^18^F-FDG uptake age-related decrease and binding

The methodology was adapted from a previous study conducted in our laboratory (Kalpouzos et al., 2009b). First, correlation analyses were conducted without covariate to identify the regions with age-related decrease in metabolism that was related to the binding scores. Positive correlations were conducted across the 34 participants by performing a multiple regression in SPM5 between preprocessed PET images for which a mask of age-related decrease in metabolism was applied and binding scores. In order to pinpoint more specific binding regions, we then entered the cognitive measures that partially mediated the effect of age on binding (revealed by the above-mentioned regression analysis; Section Behavioral data analysis) as covariates in a new model. If the brain areas that were initially correlated with binding scores ceased to be so, we would conclude that these regions were linked to the cognitive functions we entered as covariates. By contrast, if brain regions remained correlated with the binding scores, we would conclude that these regions were associated with binding *per se*.

## Results

### Behavioral results

For all results, correlations were in the expected direction, with negative correlations between age and scores and positive correlations between age and time indicating a deleterious effect of aging on scores and time, and positive correlations between scores and other scores, and negative correlations between scores and time indicating shared variance for both measurements.

#### Effect of age on binding and other cognitive functions

**Figure 2:**
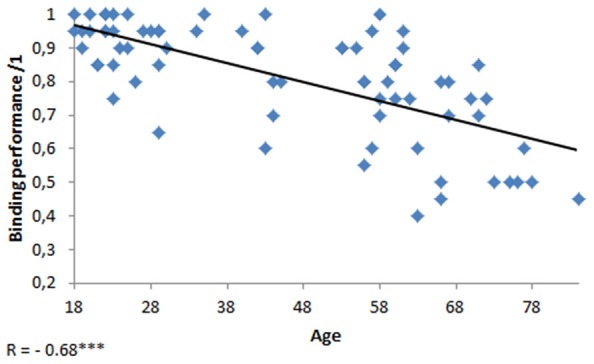
**Scatterplot of the performance at the binding task as a function of age**. ***p < 0.001.

A negative correlation between age and binding scores revealed that age had a deleterious effect on binding (*r* = −0.68, *p* < 0.001, see Figure 2). It also had a deleterious effect on inhibition (*r* = 0.49, *p* < 0.001), updating (*r* = −0.37, *p* < 0.005), the central executive (*r* = −0.36, *p* < 0.005), and processing speed (*r* = −0.46, *p* < 0.001). There was a marginal correlation between age and shifting (*r* = 0.22, *p* = 0.06).

#### Cognitive correlates of binding in working memory

Correlations are reported in Table 2. Significant correlations were found between binding scores and inhibition (*r* = −0.49, *p* < 0.001), shifting (*r* = −0.34, *p* < 0.005), updating (*r* = 0.27, *p* < 0.05), the central executive (*r* = 0.35, *p* < 0.005) and processing speed (*r* = 0.55, *p* < 0.001), with better performances on these tasks associated with better performances on the binding task.

**Table 2:** **Correlations between other cognitive functions**.

Cognitive function	Inhibition	Shifting	Updating	Central Executive
Inhibition	–			
Shifting	0.19	–		
Updating	−0.30*	−0.12	–	
Central executive	−0.30*	−0.17*	0.59***	–
Processing speed	−0.33**	−0.42***	0.26	0.24

*p < 0.05; **p < 0.01; ***p < 0.001.

#### Forward stepwise regression

The results of the forward stepwise regression are reported in Table 3. The five variables that correlated with binding performance and with age (shifting, inhibition, updating, central executive and processing speed) were included as potential explanatory variables. This analysis showed that the variance in the processing speed (first step) and inhibition (second step) measures accounted for a significant proportion of the variance in the binding measure (i.e. 40%).

**Table 3:** **Forward stepwise regression on binding accuracy with complementary cognitive scores and age for the whole sample**.

Binding		*R*^2^	Beta	*F*	*p*
Step 1		0.30		29.77	***
	Processing speed		0.55		***
Step 2		0.40		23.32	***
	Processing speed		0.43		***
	Inhibition		−0.34		***

***p < 0.001.

### Neuroimaging results

**Figure 3:**
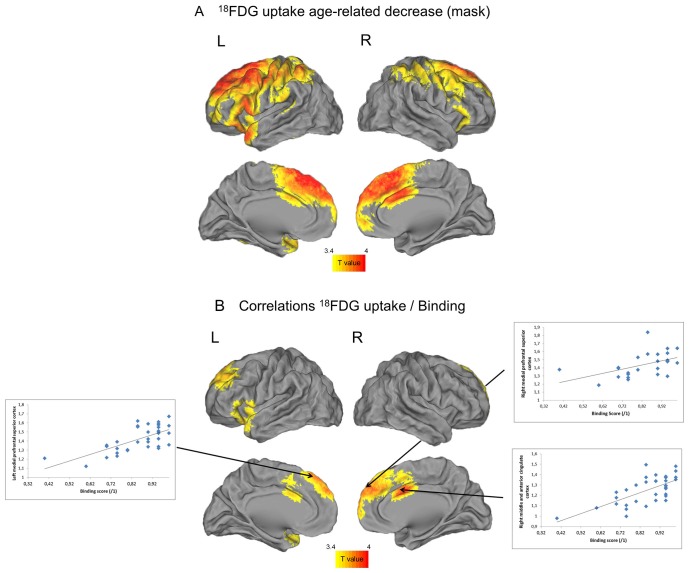
**Negative correlations between FDG uptake and age (A) and positive correlations between FDG uptake and binding limited to regions concerned by age-related changes (B)**. The threshold was significant at uncorrected *p* < 0.001.

Table 4 shows the brain regions in which ^18^F-FDG uptake significantly correlated with age (Table 4A) and binding (Table 4B). Results are illustrated in Figure 3.

**Table 4:** **Brain areas in which ^18^F-FDG uptake correlated with age (A) and binding (B)**.

MNI coordinates			*t*-value	*k*	Labeling	BA
*x*	*y*	*z*
**(A) Age**
−12	14	66	8.73	14566	Frontal mid bil	6/8/9/10/13/23/24/47
					Frontal sup bil
					Precentral bil
					Frontal sup med bil
					Postcentral L
					Supp motor area bil
					Parietal inf bil
					Frontal inf bil
					Temporal pole sup M
					Parietal sup bil
					Insula L
					Postcentral R
					Cingulate ant bil
					Cingulate mid L
					SupraMarginal L
−44	−44	−40	5.19	300	Cerebellum
54	24	−2	4.09	252	Frontal inf R	45/13
					Insula R
**(B) Binding**
6	6	28	5.08	357	Cingulate mid bil	23/24
					Cingulate ant bil
					Supp motor area bil
−6	42	52	4.71	848	Frontal sup med L	6/8
					Frontal mid L
					Frontal sup L
−30	10	−18	4.49	602	Insula L	13/47
					Temporal pole sup L
					Frontal inf L
4	66	12	4.10	563	Frontal sup med R	10/8/9
					Cingulate mid R
					Cingulate ant R

*Note: k = number of voxels within the cluster. Mid = middle. Sup = superior. Med = median. Suppl = supplementary. Inf = inferior. Ant = anterior. L = left. R = right. Bil = bilateral. BA = approximate Brodmann area*.

#### Negative correlations between ^18^F-FDG uptake and age

Negative correlations were found between age and FDG uptake mainly in frontal cortex, and extended to temporal and parietal areas (Table 4A).

#### Positive correlations between ^18^F-FDG uptake and binding

Positive correlations were found between binding performance and FDG uptake in the medial frontal, prefrontal, anterior and middle cingulate cortices on both sides, and in the left insula (Table 4B).

#### Partial correlations between ^18^F-FDG uptake and binding (with other cognitive functions partialed out)

When inhibition and processing speed were statistically controlled for, no brain area remained correlated with binding performance.

## Discussion

This study sought to explore the effect of age on the ability to bind individual features together, and to unravel the cognitive and cerebral substrates of this effect. We investigated binding using a task where individuals were asked to recognize newly created associations, and which minimized the short-term maintenance of these associations in WM. Older adults performed more poorly than younger individuals on the recognition of bound features, and the decline in binding was found to be mainly explained by an age-related decline in processing speed and executive functioning, particularly in inhibition. In accordance with a previous study conducted by Kalpouzos et al. (2009a), neuroimaging analyses showed an age-related decrease in ^18^FDG uptake in the superior, medial and inferior frontal, anterior and middle cingulate cortices, as well as in the parietal and temporal areas, bilaterally with left-sided predominance. Among these areas, the metabolism of frontal areas, the left insula and the anterior and middle cingulate cortex on both sides correlated with binding performance. Finally, when inhibition and processing speed were partialed out, no brain area remained correlated with binding accuracy.

### Behavioral results

In line with the associative-deficit hypothesis (Naveh-Benjamin, 2000) and with previous results (Chalfonte and Johnson, 1996; Mitchell et al., 2000a,b; Plancher et al., 2010), we found that age had a deleterious effect on binding. The older adults’ failure to recognize the associations partly arose from a previously demonstrated decline in executive functioning, particularly in inhibition, and processing speed (West and Alain, 2000; Rush et al., 2006; Wolf et al., 2014). These results are not surprising, as a failure of inhibitory processes is one of several hypotheses that have been put forward to explain the age-related decline in WM (Hasher and Zacks, 1988). Older adults have greater difficulty performing tasks with high WM demands, because they have difficulties to inhibit the processing of irrelevant internal and external stimuli. As a consequence, their WM is cluttered with useless material, and fewer resources are available for relevant information. They therefore experience difficulty focusing their attention on a specific resource-demanding task (Stoltzfus et al., 1996; Oberauer, 2005). The idea of a general reduction in processing speed has also been put forward to account for age-related changes in memory, and processing speed has already been identified as a mediator between age and various cognitive functions (Salthouse, 1996). In our study, cognitive slowing may have contributed to the binding decline among the older participants, especially since the presentation of the stimuli was so fast (participants had just 5 s to associate four letters with four spatial localizations in the learning phase, and only 4 s to respond in the recognition phase).

### Neuroimaging results

Our results are only partly consistent with the idea propounded in the literature that age-related decline in binding is subtended by a frontohippocampal network (Mitchell et al., 2000a; Prabhakaran et al., 2000). We found a positive correlation between age-related decrease in frontal lobe metabolism on both sides (Brodmann areas, BAs 6, 8, 10, 13 and 47) and binding accuracy. The exact role of frontal areas is not yet fully understood, but there is evidence that these brain areas are associated with executive control and monitoring of sensory inputs such as those activated along the visual dorsal and ventral paths for the representation of spatial relations (Shimamura, 2010) and allow short-term manipulation of these active representations (Mitchell et al., 2000a) and inhibitory control (Volman et al., 2011). Therefore, age-related decrease in frontal areas metabolism could be responsible for the difficulty that older adults have in encoding and maintaining associations over very short periods and possibly also in adopting efficient strategies to achieve task goals. Furthermore, when we statistically controlled for inhibition processes and processing speed, the correlation between these frontal areas and binding ceased to be significant. These findings indicate that reduced metabolism within the frontal lobes may disrupt inhibition processes and processing speed that may affect older adults’ binding ability.

Similarly, regarding the anterior and middle cingulate cortices, firstly we found that their metabolism was correlated with binding performances, and then that this did not remain true when inhibition and processing speed were statistically controlled for, suggesting that the initial correlation was also driven by inhibition and processing speed. Some studies have suggested that the anterior cingulate cortex is recruited during error detection and the resolution of cognitive conflicts between representations (Botvinick et al., 1999), and plays a role in attentional tasks (Torta and Cauda, 2011) and dual-task conditions (D’Esposito et al., 1995). An age-related reduction of anterior cingulate metabolism may have affected older individuals’ ability to detect when a letter was associated with a wrong location during the recognition phase. It may also have affected older individuals’ ability to encode an object and its location at the same time, a task imposing high attentional demands that is sometimes envisioned as a dual-task condition (Castel and Craik, 2003). Our findings also suggest that reduced metabolism of the left insula was linked to the age-related binding decline. This brain area is involved in higher-order mental processes such as the bottom-up detection of stimulus saliency across modalities (Menon and Uddin, 2010). The age-related metabolism decline in the left insula may have disrupted older participants’ ability to focus on visuospatial stimuli and hampered the detection of letters presented in different locations in the learning and recognition phases.

Interestingly, both the anterior cingulate cortex and the insula are critical areas of the salience network, whose main function is to identify the most relevant internal and external stimuli in order to guide behavior. The role of this network is to participate in the integration of bottom-up attention switching with top-down control (Menon and Uddin, 2010). This switch in attention resource allocation is expressed in the brain by switching between the default mode network and a task-specific network (Song and Tang, 2008). Thus, a dysfunction of the salience network in aging that has already been reported in a previous study (He et al., 2014) may have affected the older participants’ ability to mobilize sufficient attentional resources to focus on performing the task.

As the hippocampus was not included in the mask of age-related decrease in metabolism, which is in line with a previous study (Kalpouzos et al., 2009a), we conducted a supplementary analysis (data not shown) to test the correlations between binding performance and ^18^F-FDG uptake for the whole brain. We found that hippocampal metabolism was not correlated with binding accuracy, which is in line with a recent study showing no evidence for a critical hippocampal contribution to item-location binding in WM (Allen et al., 2014). This may be down to the characteristics of our binding task, for which the very short (1-s) interval between the encoding and retrieval of associations may not have allowed the hippocampus sufficient time to start forming long-term memory traces, contrary to what was observed in previous study featuring an 8 s interval (Piekema et al., 2006).

### Aging analogy of memory in ASD

The relationship between executive functions, WM and binding was previously reported in ASD with other paradigms. In a study by Maister et al. (2013) several relational memory tasks were used, in which participants had to produce either autobiographical memories or recall related words. The authors found that adolescents with ASD had impaired relational memory performances that were correlated with visuo-spatial WM and shifting abilities. The present study refines these observations by highlighting, in healthy subjects the contribution of inhibition and processing speed in binding processes. One might think that the general slowness that characterizes ASD (Williams et al., 2013) also participates to the binding deficit in this pathology. In addition, the impairment of inhibition observed in some patients (de Vries and Geurts, 2014) may major these difficulties. The present study also shed light on neuronal substrates that contribute to these binding deficits. Very few functional studies on WM have been conducted in ASD and they pointed out abnormalities in prefrontal and parietal cortices. We did not report correlations with parietal regions, however we have shown that the frontal lobe, the left insula and the anterior and middle part of the cingulate cortex underpinned binding decline with age. This is a very interesting result considering structural and functional abnormalities of frontal lobe and anterior cingulate cortex in ASD. Studies focused on the attentional networks reported abnormal anterior cingulate cortex activation in ASD (Agam et al., 2010; Fan et al., 2012) that may reflect neural dysfunctions in the executive control of attention. Neuroimaging investigations on WM also reported decreasing activation in prefrontal regions including the anterior cingulate during in a mental rotation task (Silk et al., 2006) and medial prefrontal regions during a one back color matching task with increasing cognitive load (Vogan et al., 2014). In light of this literature, the present data suggest that the binding deficit observed in ASD may result in part from difficulties in top-down control with reduced higher cognitive integration of complex situations.

## Conclusion

This study provides strong evidence in favor of an age-related binding decline that may partly stem from the age-related slower processing and disruption of executive processes, particularly inhibition. The older adults appeared to be disadvantaged when intentional processing was needed, which is in line with the age-related disruption of the frontal lobes. The neuroimaging analyses argued in favor of this idea and also suggest that an age-related reduction of metabolism in the left insula and in anterior and middle cingulate cortices also subtend binding decline. This pattern of results contributes to reinforce the aging analogy of memory in ASD from both a behavioral and neuronal point of view. First, processing speed is a cognitive function thought to be concerned in ASD and we could expect that it may also participate to the integration deficit. Second, the key role of the frontal lobe, the left insula and the anterior cingulate cortex into the genesis of these deficits in integration highlights the possible contribution of these areas to process complex situations in ASD.

## Conflict of interest statement

The authors declare that the research was conducted in the absence of any commercial or financial relationships that could be construed as a potential conflict of interest.

## Footnotes

^1^http://www.fil.ion.ucl.ac.uk/spm
