# Supplementary material for: Binding in working memory and frontal lobe in normal aging: is there any similarity with autism?
Source: Front Hum Neurosci. 2015 Mar 5;9:90. doi: 10.3389/fnhum.2015.00090 (PMC4362406; doi:10.3389/fnhum.2015.00090)
Supplement: Supplementary file 1 [file Table_1.DOCX]

Table 1. Correlations between other cognitive functions

| Cognitive Function | Inhibition | Shifting | Updating | Central Executive |
| --- | --- | --- | --- | --- |
| Inhibition | - |  |  |  |
| Shifting | 0.19 | - |  |  |
| Updating | -0.30* | -0.12 | - |  |
| Central executive | -0.30* | -0.17 | 0.59*** | - |
| Processing speed | -0.33** | -0.42*** | 0.26 | 0.24 |

**p* < .05; ***p* < .01; ****p* < .001
